# Supplementary material for: Real-World Therapy with Pembrolizumab: Outcomes and Surrogate Endpoints for Predicting Survival in Advanced Melanoma Patients in Germany
Source: Cancers (Basel). 2022 Apr 1;14(7):1804. doi: 10.3390/cancers14071804 (PMC8997941; doi:10.3390/cancers14071804)
Supplement: Supplementary file 1 [file cancers-14-01804-s001.zip › cancers-1643200-supplementary.pdf]

Supplementary Material

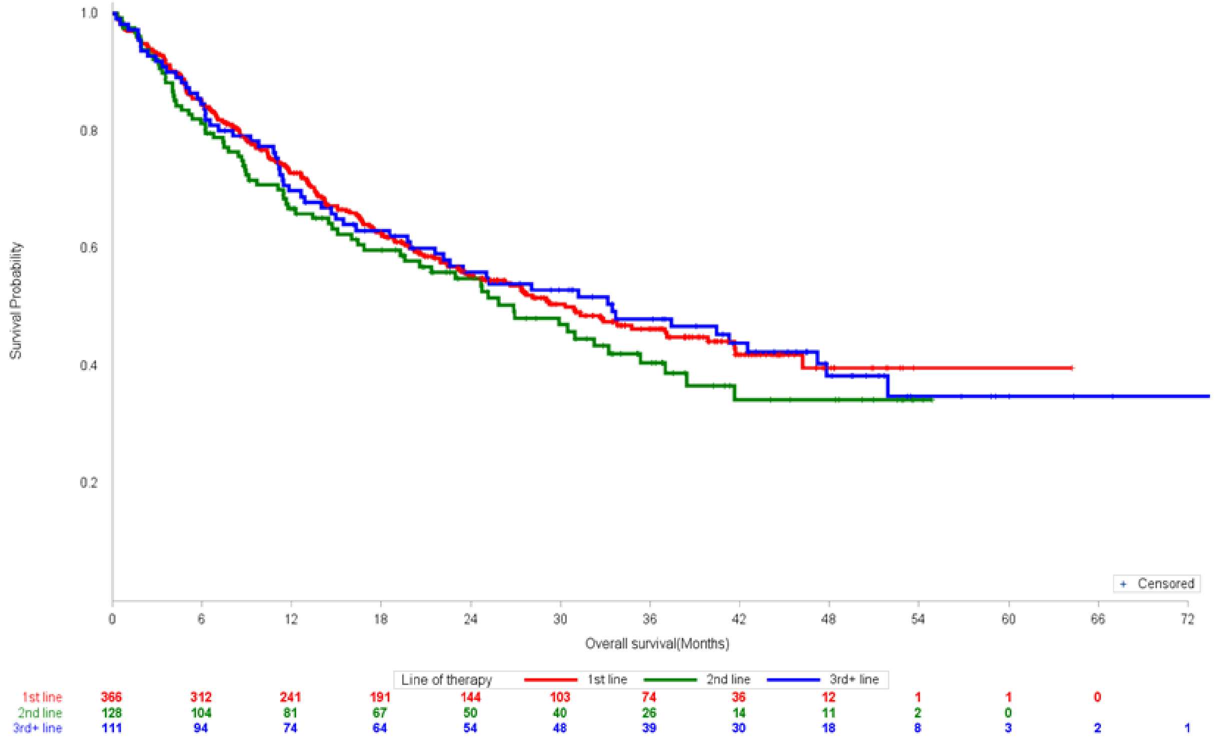

(a)

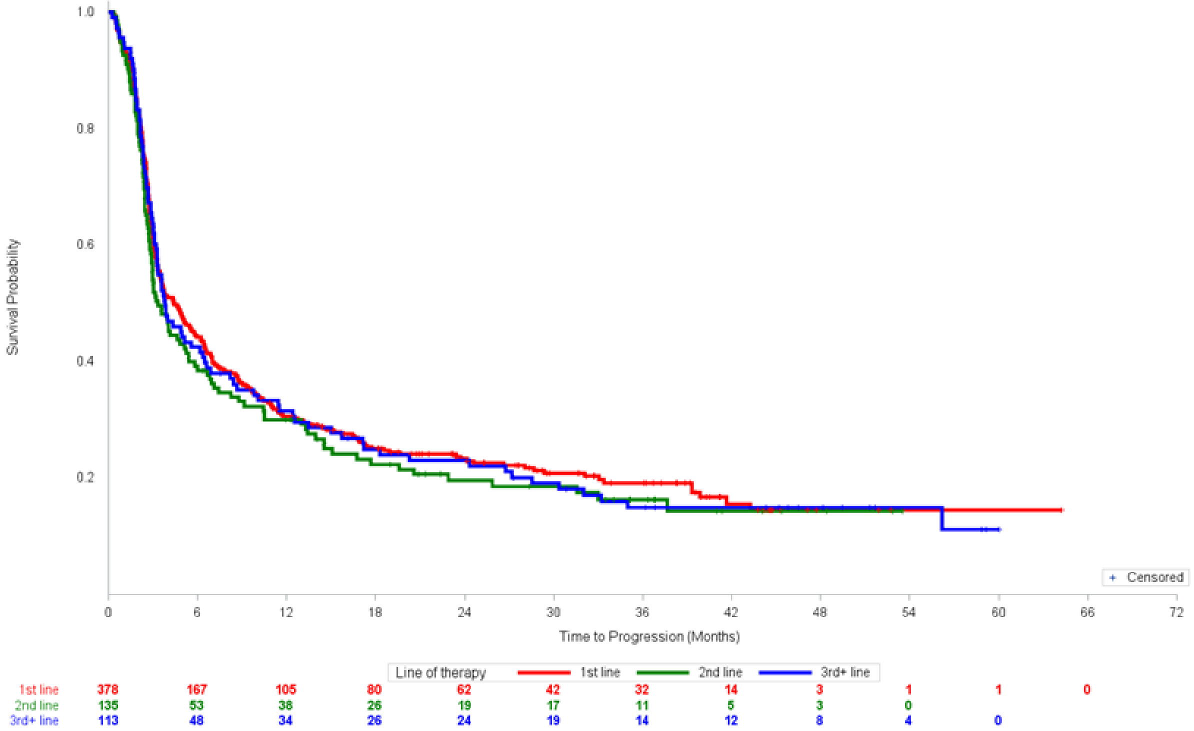

(b)

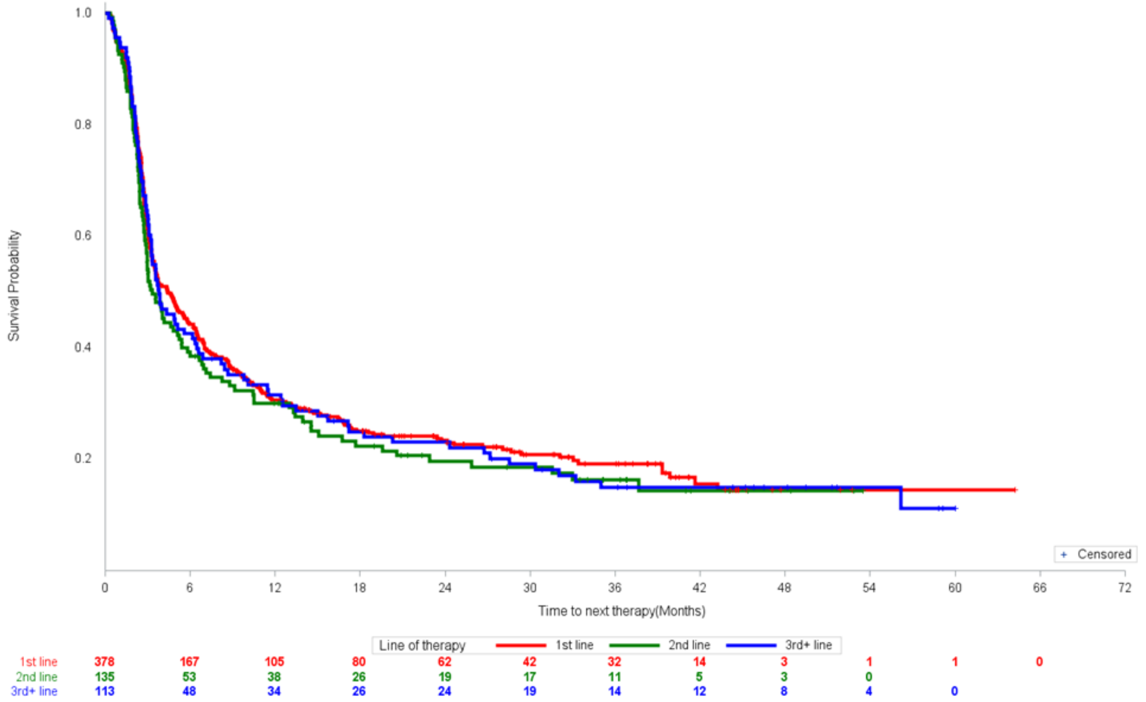

(c)

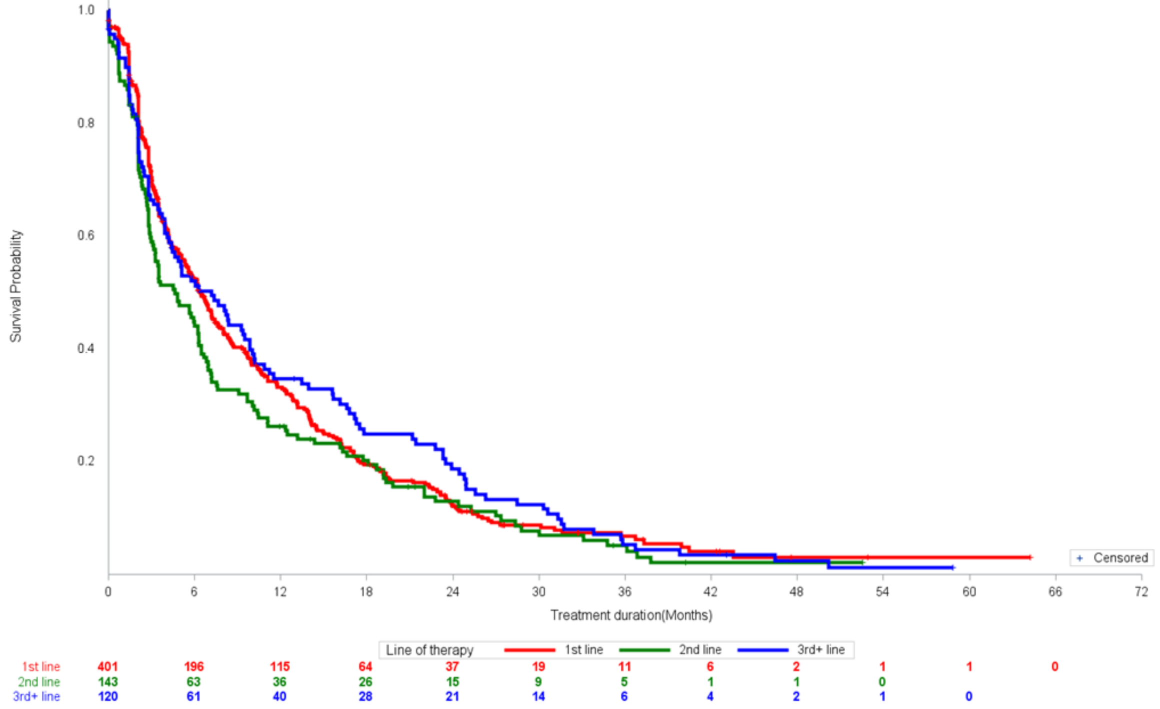

(d)

Figure S1. a-d Kaplan Meier curves showing probability estimates for rwOS (A) rwPFS (B), rwTtNT (C), and rwToT (D) of patients with advanced melanoma treated with pembrolizumab by line of therapy.

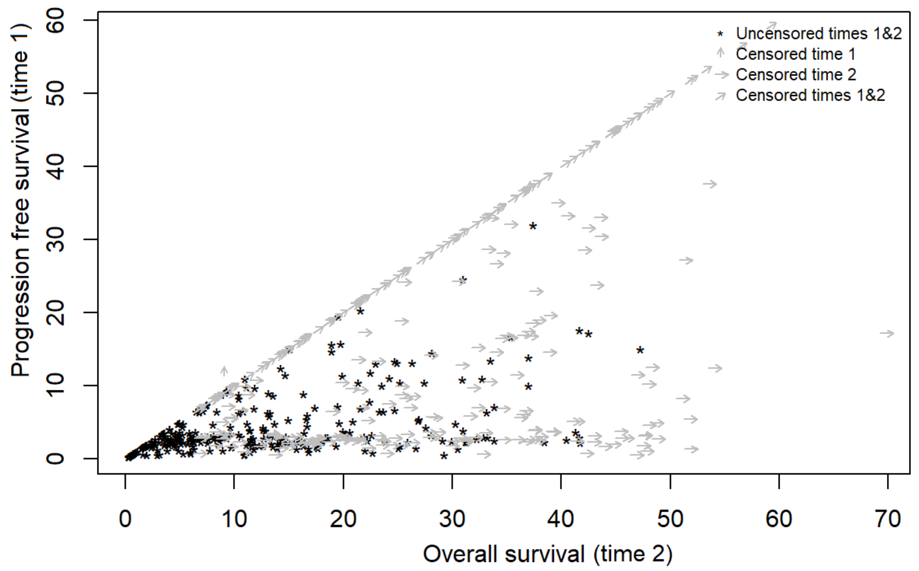
Uncensored times 1&2
Censored time 1
Censored time 2
Censored times 1&2
Progression free survival (time 1)
Overall survival (time 2)
0
10
20
30
40
50
60
70

(a)

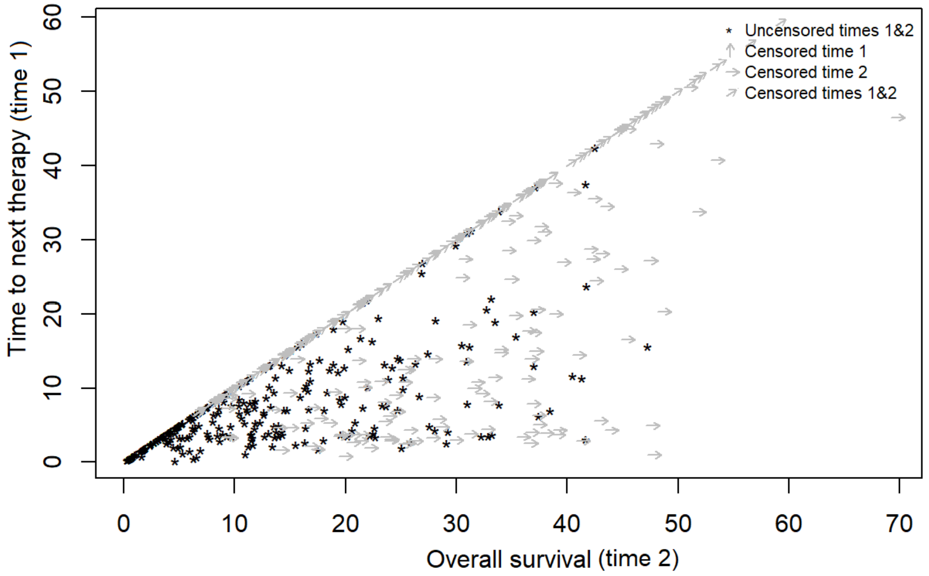
Uncensored times 1&2
Censored time 1
Censored time 2
Censored times 1&2
Time to next therapy (time 1)
Overall survival (time 2)
0
10
20
30
40
50
60
70

(b)

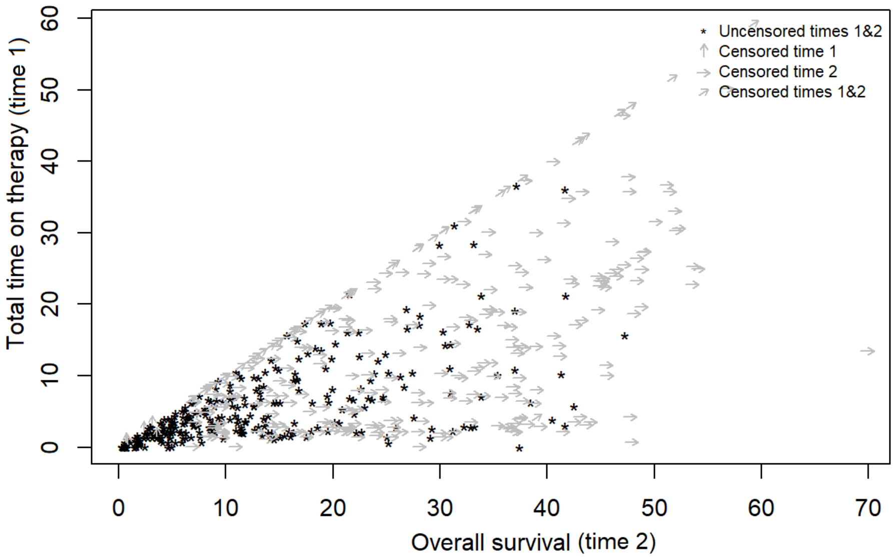

(c)

Figure S2. a-c Scatter plots depicting correlations between rwOS and rwPFS (A), rwOS and rwTtNT (B), and rwOS and rwToT (C) of patients with advanced melanoma treated with pembrolizumab, irrespective of therapy line.

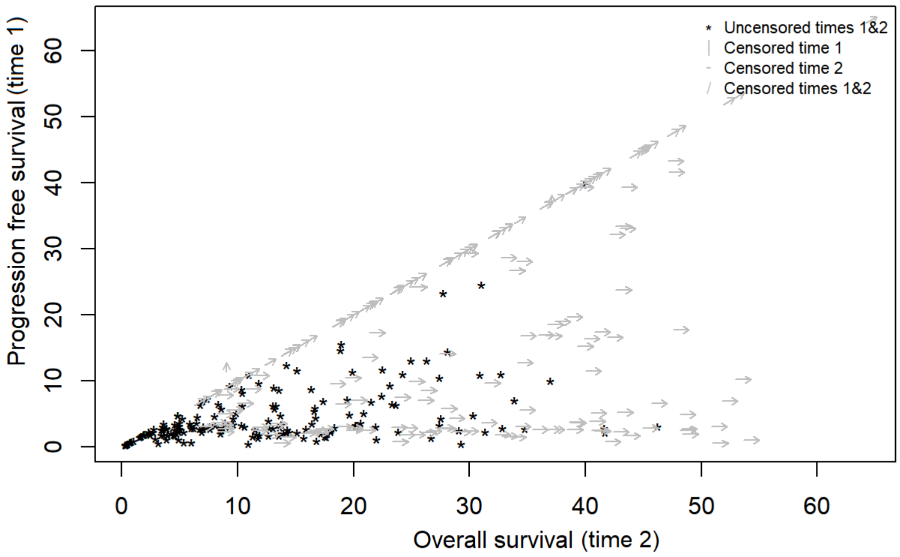
Uncensored times 1&2
Censored time 1
Censored time 2
Censored times 1&2
Progression free survival (time 1)
Overall survival (time 2)
0
10
20
30
40
50
60

(a)

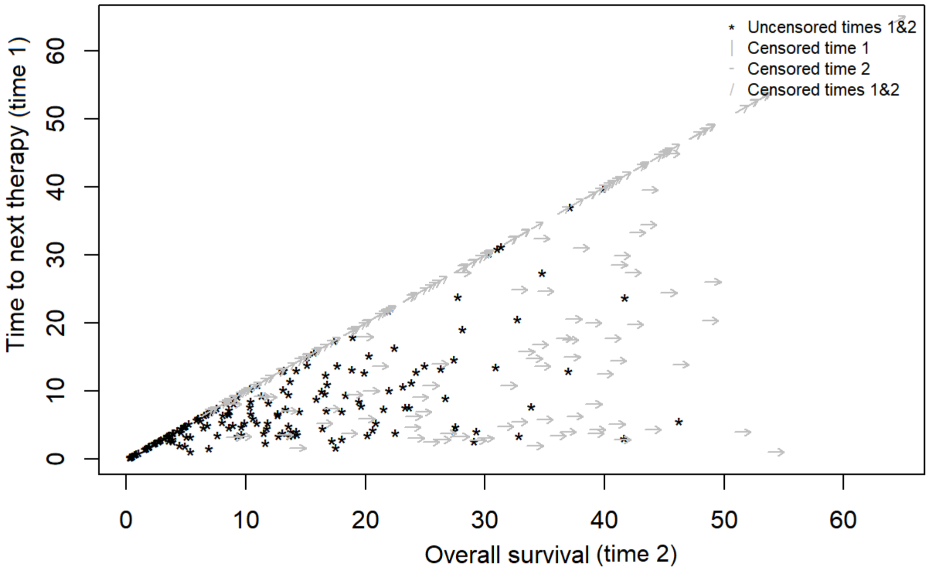
Uncensored times 1&2
Censored time 1
Censored time 2
Censored times 1&2
Time to next therapy (time 1)
Overall survival (time 2)
0
10
20
30
40
50
60

(b)

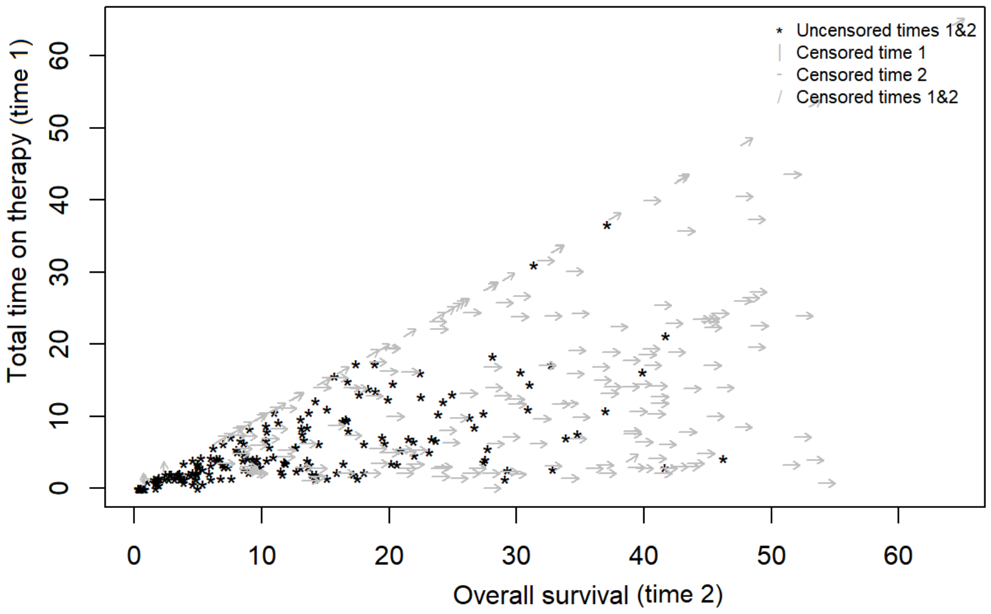

(c)

Figure S3. c Scatter plots depicting correlations between rwOS and rwPFS (A), rwOS and rwTtNT (B), and rwOS and rwToT (C) of patients with advanced melanoma treated with 1st line pembrolizumab.

Table S1. Treatment history by pembrolizumab line of therapy.

Type of anti-cancer treatment	1st line	2nd line	3+ line
**Patient count**	N=401	N=143	N=120
BRAF/MEK inhibitor n (%)	37 (9.2)	51 (35.7)	57 (47.5)
Anti-CTLA4 n (%)	31 (7.7)	63 (44.1)	73 (60.8)
Anti-CTLA4+PD-1 n (%)	72 (18.0)	15 (10.5)	5 (4.2)
Anti-PD-1 monotherapy n (%)	35 (8.7)	10 (7.0)	7 (5.8)
Other* n(%)	59 (14.7)	52 (36.4)	81 (6.8)

* includes interferons, chemotherapies and combinations of therapies not specifically included in above categories.

Table S2. Kaplan-Meier estimate for median rwOS and rwPFS (from index date), overall and by line of therapy.

**Estimate**	**Overall**	**1st line**	**2nd line**	**3+ line**
**Survival time rwOS**	N=605	N=366	N=128	N=111
Censored observations n (%)	299 (49.4)	191 (52.2)	58 (45.3)	50 (45.1)
Events n (%)	306 (50.6)	175 (47.8)	70 (54.7)	61 (55.0)
Median survival time (95% CI), months	30.5 (25.0, 35.4)	30.9 (23.8, 41.6)	26.9 (19.3, 37.0)	33.5 (21.6, 47.8)
**Survival rate rwOS, % (SE)**				
6 months	84.3 (1.5)	85.2 (1.9)	81.3 (3.4)	84.7 (3.4)
12 months	71.0 (1.9)	72.9 (2.4)	66.8 (4.2)	69.8 (4.4)
18 months	62.2 (2.0)	62.8 (2.6)	59.8 (4.4)	63.1 (4.7)
24 months	55.4 (2.1)	55.3 (2.8)	54.9 (4.6)	56.0 (4.8)
**Real-world progression free time**	N=626	N=378	N=135	N=113
Censored observations n (%)	124 (19.8)	81 (21.4)	25 (18.5)	18 (15.9)
Events n (%)	502 (80.2)	297 (78.6)	110 (81.5)	95 (84.1)
Median survival time (95% CI), months	3.9 (3.5, 4.9)	4.4 (3.5, 5.8)	3.35 (2.9, 5.2)	3.81 (3.22, 6.3)
**Real-world progression free rate, % (SE)**				
6 months	42.8 (2.0)	44.2 (2.6)	39.3 (4.2)	42.5 (4.7)
12 months	30.7 (1.9)	30.6 (2.4)	30.0 (4.0)	31.5 (4.4)
18 months	24.5 (1.8)	25.1 (2.3)	22.4 (3.7)	24.9 (4.1)
24 months	22.5 (1.7)	23.4 (2.3)	19.6 (3.6)	23.0 (4.0)

Note. NR: not reached; rwOS: real-world overall survival; rwPFS: real-world progression free survival.

Table S3. Kaplan-Meier estimate for median rwTtNT (from index date) and on treatment rates, overall and by line of therapy.

Estimate	Overall	1st line	2nd line	3+ line
**Median rwTtNT**	N=664	N=378	N=135	N=113
Censored observations, n (%)	230 (34.6)	148 (36.9)	43 (30.1)	39 (32.5)
Events, n (%)	434 (65.4)	253 (63.1)	100 (69.9)	81 (67.5)
Median rwTtNT (95% CI), months	10.7 (9.0, 12.9)	10.8 (9.0, 13.6)	8.9 (7.0, 13.4)	12.5 (8.2, 18.5)
**Estimates for rates of not on subsequent treatment, % (SE)**				
6 months	65.5 (1.9)	66.8 (2.4)	59.8 (4.2)	67.8 (4.4)
12 months	47.6 (2.0)	48.4 (2.6)	42.9 (4.3)	50.9 (4.8)
18 months	37.5 (2.0)	37.8 (2.6)	33.8 (4.3)	41.1 (4.7)
24 months	33.8 (2.0)	34.1 (2.6)	32.0 (4.2)	35.1 (4.6)

Note. CI: confidence interval; rwTtNT: real-world time to next treatment; SE: standard error.

Table S4. Kaplan-Meier estimate for median rwToT (from index date), overall and by line of therapy.

Estimate	Overall	1st line	2nd line	3+ line
**Median rwToT**	N=664	N=401	N=143	N=120
Censored observations, n (%)	78 (11.7)	62 (15.5)	10 (7.0)	6 (5.0)
Events, n (%)	586 (88.3)	339 (84.5)	133 (93.0)	114 (95.0)
Median rwToT (95% CI), months	6.2 (5.1, 6.8)	6.4 (5.3, 7.4)	4.6 (3.1, 6.3)	7.1 (4.2, 9.9)
**Estimates for restricted mean rwToT (SE), months**				
At 24 months	9.3 (0.3)	9.4 (0.4)	8.2 (0.7)	10.2 (0.8)
At 30 months	9.9 (0.4)	10.0 (0.5)	8.8 (0.8)	11.0 (1.0)
At 36 months	10.4 (0.4)	10.5 (0.5)	9.2 (0.9)	11.5 (1.1)
**Estimates for on treatment rates, % (SE)**				
12 months	31.9 (1.9)	33.1 (2.5)	26.3 (3.7)	34.7 (4.4)
24 months	13.6 (1.4)	12.1 (1.8)	12.9 (2.9)	18.7 (3.6)
30 months	9.2 (1.2)	8.8 (1.6)	7.8 (2.4)	12.5 (3.1)
36 months	5.7 (1.1)	6.8 (1.5)	5.2 (2.0)	5.3 (2.1)

Note. CI: confidence interval; rwToT: real-world time on treatment; SE: standard error.

Table S5. Real-world Tumor Response Rate (rwTRR) and real-world Tumor Control Rate (rwTCR), overall and by line of therapy.

Estimate	Overall	1st line	2nd line	3+ line
**rwTRR, % (95% CI)**				
Missing	7.2 (5.3, 9.2)	8.7 (6.0, 11.5)	4.9 (1.4, 8.4)	5.0 (1.1, 8.9)
Response	24.4 (21.1, 27.7)	24.9 (20.7, 29.2)	21.0 (14.3, 27.7)	26.7 (18.8, 34.6)
Non-response	68.4 (64.8, 71.9)	66.3 (61.7, 71.0)	74.1 (66.9, 81.3)	68.3 (60.0, 76.7)
**rwTCR, % (95% CI)**				
Missing	7.2 (5.3, 9.2)	8.7 (6.0, 11.5)	4.9 (1.4, 8.4)	5.0 (1.1, 8.9)
Control	40.2 (36.5, 43.9)	43.4 (38.5, 48.2)	35.0 (27.1, 42.8)	35.8 (27.3, 44.4)
Non-control	52.6 (48.8, 56.4)	47.9 (43.0, 52.8)	60.1 (52.1, 68.2)	59.2 (50.4, 68.0)

Note. CI: confidence interval; rwTCR: real-world tumor control rate; rwTRR: real-world tumor response rate.
